# Disrupted Coupling Between the Spontaneous Fluctuation and Functional Connectivity in Idiopathic Generalized Epilepsy

**DOI:** 10.3389/fneur.2018.00838

**Published:** 2018-10-05

**Authors:** Xiaoyan Jia, Shuai Ma, Sisi Jiang, Honbin Sun, Debo Dong, Xuebin Chang, Qiong Zhu, Dezhong Yao, Liang Yu, Cheng Luo

**Affiliations:** ^1^The Clinical Hospital of Chengdu Brain Science Institute, MOE Key Lab for Neuroinformation, Center for Information in Medicine, High-Field Magnetic Resonance Brain Imaging Key Laboratory of Sichuan Province, School of Life Science and Technology, University of Electronic Science and Technology of China, Chengdu, China; ^2^Neurology Department, Sichuan Provincial People's Hospital, The Affiliated Hospital of University of Electronic Science and Technology of China, Chengdu, China

**Keywords:** idiopathic generalized epilepsy, coupling, functional magnetic resonance imaging, amplitude of low frequency fluctuation, functional connectivity density

## Abstract

**Purpose:** The purpose of this study was to comprehensively evaluate alterations of resting-state spontaneous brain activity in patients with idiopathic generalized epilepsy (IGE) and its subgroups [juvenile myoclonic epilepsy (JME) and generalized tonic-clonic seizures (GTCS)].

**Methods:** Resting state functional magnetic resonance imaging (fMRI) data were acquired from 60 patients with IGE and 60 healthy controls (HCs). Amplitude of low frequency fluctuation (ALFF), global functional connectivity density (gFCD), local FCD (lFCD), and long range FCD (lrFCD) were used to evaluate spontaneous brain activity in the whole brain. Moreover, the coupling between ALFF and FCDs (gFCD, lFCD, and lrFCD) was analyzed on both voxel-wise and subject-wise levels. Two-sample *t*-tests were used to analyze the difference in ALFF, FCDs and coupling on a subject-wise level between the two groups. Nonparametric permutation tests were used to evaluate differences in coupling on a voxel-wise level.

**Key findings:** Patients with IGE and its subgroups showed reduced ALFF, gFCD and lrFCD in posterior regions of the default mode network (DMN). In addition, decreased ALFF and increased coupling with FCD were found in the cerebellum, while decreased coupling was observed in the bilateral pre- and postcentral gyrus in IGE compared with the coupling in HCs. Similar findings were found in the analysis between each of the two subgroups of IGE (JME and GTCS) and HCs, and JME patients had increased coupling in the cerebellum and bilateral middle occipital gyrus compared with coupling in the GTCS patients.

**Significance:** This study demonstrated a multifactor abnormality of the DMN in IGE and emphasized that the abnormality in the cerebellum was associated with dysfunctional motor symptoms during seizures and might participate in the regulation of GSWDs in IGE.

## Introduction

Idiopathic generalized epilepsy (IGE) is characterized by widespread generalized epileptic discharges, including generalized tonic-clonic, absence, and myoclonic seizures (1). As a main subtype of IGE, generalized tonic-clonic seizures (GTCS) is often followed by irregular clonic jerking (2). Using scalp electroencephalography (EEG), generalized spike-and-wave discharges (GSWDs) at 2.5–5 Hz are observed in GTCS. Juvenile myoclonic epilepsy (JME), another subtype of IGE, often presents with the onset of seizures predominantly between 12 and 18 years of age. JME is characterized clinically by myoclonic jerks after awakening, tonic-clonic seizures, and an EEG with 3–4 Hz poly-spikes and GSWDs with fronto-central predominance (3). Accumulated evidence from previous studies has demonstrated the participation of the thalamus and widespread cortical regions in the generation and propagation of epileptic activity (4–6). In recent years, non-invasive neuroimaging techniques such as functional magnetic resonance imaging (fMRI) provided helpful tools to uncover the potential mechanisms of IGE (7–9). For example, functional connectivity (FC) analysis shows that generalized seizures are related to bilateral distributed networks (10). Recently, some researchers have proposed that the coupling of fMRI features would provide a complementary strategy to evaluate the state of the human brain (11). Thus, we analyzed the brain dysfunction associated with IGE and its subgroups using a coupling of fMRI features.

Amplitude of low frequency fluctuation (ALFF) and functional connectivity density (FCD) are fundamental features of fMRI that represent voxel-level local neural activity and functional integration, respectively (12, 13). Many studies have suggested that ALFF of fMRI signals are closely related to the spontaneous neuronal electrophysiological activity such as delta or alpha rhythm (14, 15). In previous studies, significant differences in ALFF were revealed in the thalamus and prefrontal cortex in patients with IGE (16, 17). Meanwhile, FCD reflects communication between different regions. Tomasi and his colleagues suggested that higher glucose metabolism in hub regions supports a higher communication rate in these regions (12). In GTCS, alterations of the functional hub was primarily located in the sensorimotor network (SMN) and the default mode network (DMN) (18). Furthermore, significantly increased long range FCD (lrFCD) was demonstrated in sensorimotor areas, and decreased lrFCD was demonstrated in the prefrontal, inferior parietal and temporal cortices in GTCS (19). These studies separately revealed changes in ALFF and FCD in patients with IGE. Recent evidence indicated that combining ALFF and FCD might improve the capability for focusing detection and comprehensively revealing the intrinsic brain network dysfunction (11, 20), although a specific neuronal processes is difficult to find to match the coupling between two resting state features. The decreased coupling of amplitude and connectivity in mesial temporal regions and increased coupling in posterior regions of the DMN have been revealed in patients with temporal lobe epilepsy (11). Decreased coupling between ALFF and FCD in the hippocampus and parahippocampus was observed in systemic lupus erythematosus patients without overt neuropsychiatric symptoms (20). In addition, a sliding window approach has been used to evaluate the dynamic ALFF and dynamic FC in patients with schizophrenia, and altered coupling between dynamic ALFF and FC associated with schizophrenia were revealed, suggesting a disruption of the adaptive network property (21). However, there is no study that has combined ALFF and FCD to comprehensively evaluate the altered resting-state spontaneous brain activity in patients with IGE. We hypothesized that altered ALFF and FCD would be observed in IGE and its subgroup. Additionally, the coupling of ALFF and FCD might be altered in some specific regions related to motor manifestations such as sensorimotor cortex and cerebellum.

The current study evaluated alterations in ALFF and FCDs [global FCD (gFCD), local FCD (lFCD), and lrFCD] separately in patients with IGE and its subgroups and healthy controls (HCs). Moreover, the alteration of coupling between ALFF and FCDs was also evaluated in patients and HCs. Additionally, correlation analyses between functional features (ALFF, gFCD, lFCD, and lrFCD) and clinical factors in patients were performed.

## Materials and methods

### Subjects

A total of 60 patients with IGE (31 females; mean age = 25.47 ± 8.96 years; mean disease duration = 10.35 ± 9.13 years) from the Affiliated Hospital of University of Electronic Science and Technology of China were recruited. Diagnosis was established according to the clinical and seizure semiology information consistent with the International League Against Epilepsy (ILAE) guidelines (22). All patients were treated with antiepileptic drugs (AEDs). The detail information is demonstrated in Supplementary Table 1 in the supporting information. The seizure frequency is demonstrated in Supplementary Table 2 in the supporting information. These IGE patients were divided into two subgroups according to their clinical epilepsy syndromes, including 28 GTCS only (13 females; mean age = 26.96 ± 10.79 years; mean disease duration = 9.57 ± 11.01 years) and 32 JME only (18 females; mean age = 24.16 ± 6.91 years; mean disease duration = 11.03 ± 7.22 years). Sixty gender- and age-matched healthy subjects (32 female; mean age = 25.88 ± 8.09 years) were recruited as the HCs group. All detail information are shown in Table 1. All controls had no history of neurological disorder or psychiatric illness and no gross abnormalities on brain MRI images. Gender and age were considered covariates in the subsequent statistical comparisons. Written informed consent was obtained from each subject. This study was approved by the ethical committee of the University of Electronic Science and Technology of China according to the standards of the Declaration of Helsinki.

**Table 1:** Characteristics of patients and HC.

**Characteristics**	**IGE** **(*n* = 60)** **Mean ± SD**	**JME** **(*n* = 32)** **Mean ± SD**	**GTCS** **(n = 28)** **Mean ± SD**	**HC** **(n = 60)** **Mean ± SD**	**P1**	**P2**	**P3**	**P4**
Age	25.47 ± 8.96	24.16 ± 6.91	26.96 ± 10.79	25.88 ± 8.09	0.86^a^	0.84^a^	0.30^a^	0.99^a^
Gender (female/male)	31/29	18/14	13/15	32/28	0.855^b^	0.789^b^	0.546^b^	0.448^b^
Duration (year)	10.35 ± 9.13	11.03 ± 7.22	9.57 ± 11.01	–	–	–	–	0.28^a^

P1 represent the test results of IGE and HC.

P2 represent the test results of JME and HC.

P3 represent the test results of GTCS and HC;

P4 represent the test results of GTCS and JME.

a The p-value obtained by Wald-Wolfowitz run test.

b *The p-value obtained by chi-square test*.

### Data acquisition

The resting-state functional data were acquired using gradient echo-planar imaging (EPI) sequences in a 3T GE scanner with an eight-channel-phased array head coil (MR750; GE Discovery, Milwaukee, WI) at the MRI research center of the University of Electronic Science and Technology of China. The imaging parameters were as following: repetition time = 2,000 ms, echo time = 30 ms, flip angle = 90°, field of view = 24 × 24 cm^2^, matrix size = 64 × 64, and slice thickness = 4 mm with 0.4 mm gap and 255 volumes were collected in each run. Axial anatomical T1-weighted images were acquired using a 3-dimensional fast spoiled gradient echo (T1-3D FSPGR) sequence [repetition time = 6.008 ms, echo time = 1.984 ms, flip angle = 90°, matrix size = 256 × 256, field of view = 25.6 × 25.6 cm^2^, and slice thickness = 1 mm (no gap)] to generate 152 slices. During fMRI acquisition, all subjects were instructed to have their eyes closed, move as little as possible, stay awake and not think about anything in particular. After scanning, subjects were asked about their experiences during the scan, including sleep, comfort, and noise.

### Data preprocessing

Preprocessing of fMRI data was completed using the SPM12 software package (statistical parametric mapping available at: http://www.fil.ion.ucl.ac.uk/spm). (1) The first 5 volumes were excluded to ensure a steady magnetic field. (2) Slice timing was corrected. (3) There was realignment to the remaining 250 images. No rotation or translation parameters in any given data set exceeded 2 mm or 2°. Moreover, we evaluated frame-wise displacement (FD) in all groups using the following formula: $\text{FD}=\left(1/\left(M-1\right)\right)\sum_{i=2}^{M}\left(\left|\Delta d_{x_{i}}\right|+\left|\Delta d_{y_{i}}\right|+\left|\Delta d_{xz_{i}}\right|+\left|\Delta d_{l_{i}}\right|+\left|\Delta d_{m_{i}}\right|+\left|\Delta d_{n_{i}}\right|\right)$, where *M* is the length of the time course (*M* = 250 here), *x*_*i*_, *y*_*i*_, and *z*_*i*_ are translations in the *x, y*, and *z* directions at the *i*th time point, Δ*d*_*x*_*i*__ = *x*_*i*_ − *x*_*i*−1_, and Δ*d*_*y*_*i*__ and Δ*d*_*z*_*i*__ is calculated using similar formulas. Meanwhile, *l*_*i*_, *m*_*i*_, and *n*_*i*_ are rotations in the *x, y*, and *z* directions at the *i*th time point and Δ*d*_*l*_*i*__ = 50 × π × (*l*_*i*_ − *l*_*i*−1_)/180 (similar formulas were used for Δ*d*_*m*_*i*__ and Δ*d*_*n*_*i*__). FD was regressed as a covariate in the subsequent statistical calculation. (4) The realigned images were spatially normalized to the Montreal Neurological Institute (MNI) template using a 12-parameter affine transformation and resliced with voxel size of 3 × 3 × 3 mm. Then, the data went through separate and additional preprocessing for the calculation of ALFF and FCDs. For the preprocessing for ALFF, all spatially normalized data were smoothed with an 8 mm full width at half maximum (FWHM) Gaussian kernel. For the preprocessing of FCDs, in order to avoid artificially introducing local spatial correlations, images were not smoothed after they were spatially normalized (23). Then, we regressed out the nuisance signals, including 6 head motion parameters, the white matter signal, the cerebral spinal fluid signal and the linear drift signal. We did not regress the global signal of the brain because global signal regression probably leads to anticorrelations in fMRI data (24). Finally, bandpass filtering (0.01–0.08 Hz) was conducted on the time series of each voxel.

### ALFF analysis

ALFF analysis was conducted using REST software (http://restfmri.net/forum/REST_V1.8) (25). The time series was converted to the frequency domain by means of using the fast Fourier transform (FFT) to obtain the power spectrum. Then, the power spectrum was square-rooted and averaged across 0.01–0.08 Hz at each voxel. These results were defined as ALFF (13). Finally, we divided the ALFF value of each voxel by the global mean value for each participant.

### FCD analysis

The lFCD and gFCD were calculated to evaluate the strength of voxel-wise FC (12). FC was evaluated using Pearson's correlation analyses. The threshold of the correlation coefficient was set as 0.6, similar to our previous studies (26, 27).

The steps of the calculation of lFCD and gFCD included the following (12). First, the lFCD at a given voxel *x*_0_ was calculated as the number of FC above threshold between voxel *x*_0_ and other voxels within its local cluster. A 3-dimensional searching algorithm developed in Interactive Data Language (IDL) was used to determine the local cluster of *x*_0_. If voxel *x*_i_ is adjacent to a voxel which belongs to the list of neighbors of *x*_0_ and the correlation value between *x*_0_ and *x*_i_ exceeded the correlation threshold (*r* = 0.6), the voxel *x*_i_ was added to the list of neighbors of *x*_0_. This calculation was repeated for next voxel which was on the list of neighbors. The computation of gFCD was similar to the calculation of lFCD; however, it was not restricted within its local cluster but extended to all other voxels in the gray matter mask. The calculation of lFCD and gFCD was repeated for all voxels within the gray matter mask. In this manner, the lFCD and gFCD maps of all subjects were obtained. In addition, the lrFCD was generated by subtracting the lFCD from the gFCD (28). The individual FCD map was normalized by dividing by the mean value across voxel in the gray matter mask. Then, all FCD maps were smoothed with 8 mm FWHM Gaussian kernel.

### Coupling between ALFF and FCDs

FCD could indicate brain functional integration, and ALFF can reflect the brain functional segregation (29). Therefore, the coupling between ALFF and FCDs could comprehensively and synergistically expose the dysfunction in patients with IGE and its subgroups. Based on previous studies (11, 20), Pearson correlation was used to evaluate the coupling between ALFF and FCDs. The Pearson correlations between ALFF and FCDs were calculated both voxel-wise and subject-wise in the gray matter mask which was generated by averaging the gray matter segmentations of T1 weight scans from all subjects (30). The gray matter mask was created by SPM masking toolbox (http://www0.cs.ucl.ac.uk/staff/g.ridgway/masking/).

For a given voxel *v*_0_, the correlation (voxel-wise) was calculated across subjects. First, the ALFF value and the lFCD value at same voxel *v*_0_ were extracted for one group. The Pearson correlation between one-dimensional vectors (across subjects) of ALFF and lFCD was calculated to obtain the voxel-wise correlation value at voxel *v*_0_. The calculation was repeated for all voxels within the gray matter mask to obtain one voxel-wise correlation map. In addition, the same steps were carried out for the coupling between ALFF and gFCD and the coupling between ALFF and lrFCD. All of these calculations were performed for the HCs and patient groups.

The subject-wise correlations between ALFF and FCDs was computed for each subject. ALFF and lFCD maps within the gray matter mask of each subject were reshaped as a one-dimensional vector separately. Then, Pearson correlations between one-dimensional vectors of ALFF and lFCD were calculated to obtain the subject-wise coupling in each subject. The same calculation was applied to the subject-wise correlation between ALFF and gFCD and between ALFF and lrFCD. These three types of subject-wise coupling were calculated in all subjects.

### Statistical analysis

We performed separate one-sample *t*-tests for ALFF, gFCD, lFCD, and lrFCD maps to evaluate the distribution maps of these features in all groups. Then, in order to assess alterations of the above four imaging features in the IGE group relative to the maps in the HCs group, a two-sample *t*-test was carried out within the gray matter mask by threshold-free cluster enhancement (TFCE) which was implemented in FSL (31) with *p* < 0.05 family-wise error corrected. Furthermore, we divided the patient group into JME and GTCS subgroups and performed the comparisons between the JME, GTCS and HCs groups to assess differences for each type of patient. Finally, Pearson correlation analyses were calculated between the clinical factors in patients and the above four imaging indicators for brain regions with significant differences between groups.

For the coupling analyses, a two-sample *t*-test was conducted on the Fisher's *r*-to-*z* transformed results of the subject-wise correlation between groups. On voxel-wise level, non-parametric permutation tests with 10,000 repetitions were used to evaluate the difference in coupling between ALFF and gFCD, lFCD, and lrFCD in regions with significant correlations between ALFF and FCDs in both groups. The significance level of the non-parametric permutation test was set as a one-tailed *p* < 0.01 (uncorrected) and a cluster size of at least 30 voxels was used. All neuroanatomical localization are based on Harmmers et al. (32).

## Results

### Imaging feature comparison between patients and HCs

One-sample *t*-test results are shown in Supplementary Figure 1 in the supporting information and show the spatial distribution of imaging parameters in all groups.

**Figure 1:**
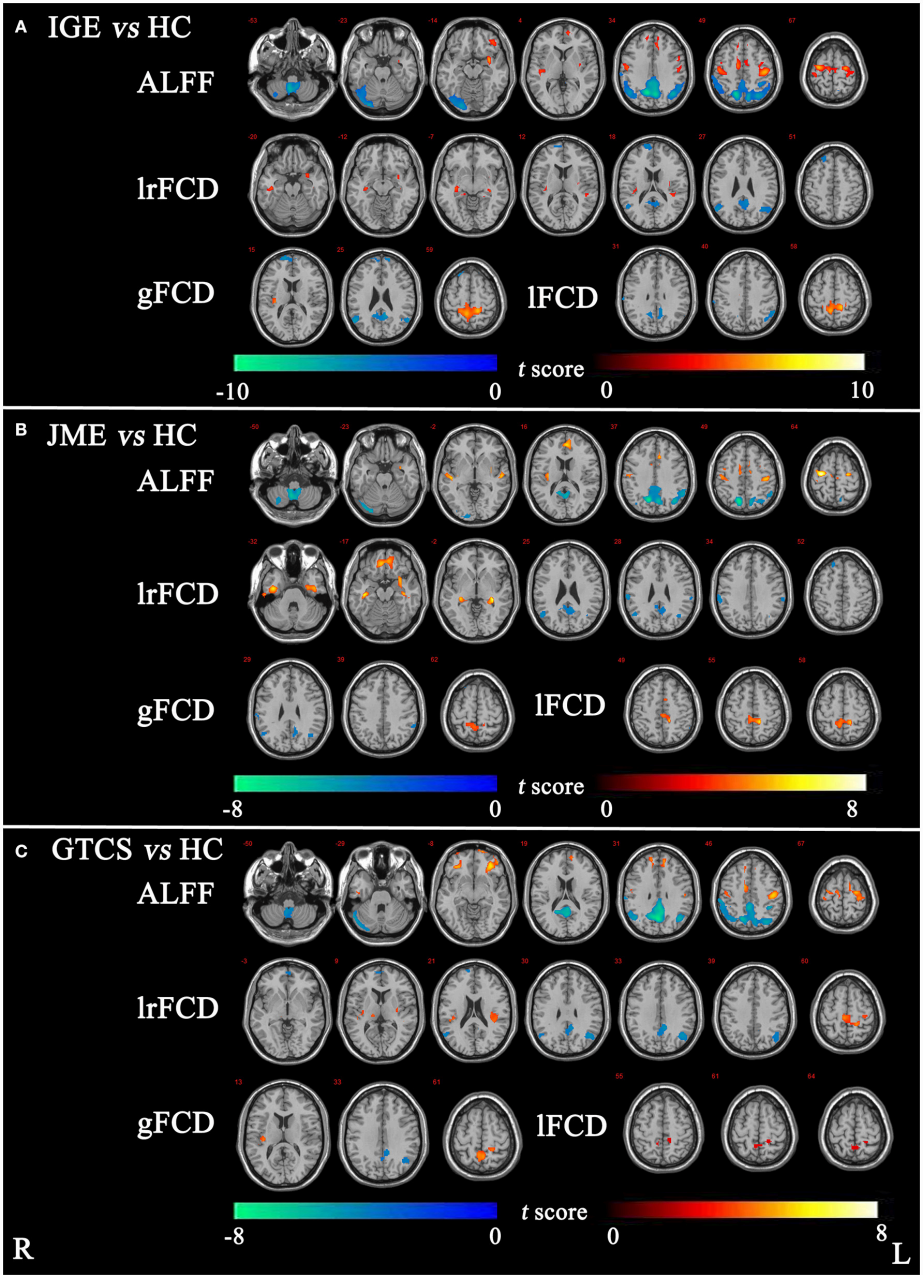
**(A)** The significant differences of ALFF and FCDs between IGE and HC. **(B)** The significant differences of ALFF and FCDs between JME and HC. **(C)** The significant differences of ALFF and FCDs between GTCS and HC (Threshold-Free Cluster Enhancement, *p* < 0.05). The significant increased regions were showed with positive *t*-value and significant decreased regions with negative *t*-value. R, Right; L, Left.

The significant differences in ALFF and FCDs between IGE and HCs (TFCE, *p* < 0.05) are shown in Figure 1A and Table 2. Compared with the HCs group, the IGE group had a significantly increased ALFF in the right precentral gyrus and a significantly decreased ALFF in the right superior parietal gyrus and right cerebellum. The significantly increased lrFCD were located in the left posterior temporal lobe and right middle and inferior temporal gyrus and the significantly decreased lrFCD were found in the right inferolateral remainder of parietal lobe, left cingulate gyrus of posterior part and right superior frontal gyrus. The significantly increased gFCD was located in the left superior parietal gyrus, and the significantly decreased gFCD were located in the left inferolateral remainder of parietal lobe, left cingulate gyrus of posterior part and right superior frontal gyrus. The significantly increased lFCD were located in the left superior parietal gyrus, and the significantly decreased lFCD were located in the right superior parietal gyrus and left inferolateral remainder of parietal lobe.

**Table 2:** Significant differences of ALFF and FCDs between patients with IGE and HC.

**Parameter** **(IGE > HC)**	**Brain regions (Harmmers atlas)**	**MNI coordinates**			**Cluster size**	**Peak *t*-value**
		**X**	**Y**	**Z**		
ALFF	Right precentral gyrus	30	−12	63	2,611	7.1707
	Right superior parietal gyrus	12	−66	36	3,600	−9.7908
	Right cerebellum	6	−54	−57	439	−7.1295
	Right cerebellum	27	−90	−27	1,004	−6.6404
lrFCD	Left posterior temporal lobe	−33	−33	−3	98	5.4034
	Right middle and inferior temporal gyrus	42	−24	−21	208	5.2008
	Right inferolateral remainder of parietal lobe	51	−63	21	200	−5.8661
	Left cingulate gyrus, posterior part	−9	−51	27	567	−5.4741
	Right superior frontal gyrus	12	63	15	302	−5.4282
gFCD	Left superior parietal gyrus	−15	−42	54	717	5.6583
	Left inferolateral remainder of parietal lobe	−36	−66	30	490	−5.2462
	Left cingulate gyrus, posterior part	−9	−51	27	417	−5.0217
	Right superior frontal gyrus	15	63	15	389	−4.7921
lFCD	Left superior parietal gyrus	−15	−42	54	797	5.8142
	Right superior parietal gyrus	18	−51	21	104	−4.8566
	Left inferolateral remainder of parietal lobe	−60	−51	39	180	−4.2637

*MNI, Montreal Neurological Institute*.

The significant differences in ALFF and FCDs between the JME and HCs groups (TFCE, *p* < 0.05) are shown in Figure 1B and Table 3. Compared with the HCs group, the JME group showed significantly increased ALFF in the bilateral precentral gyrus and significantly decreased ALFF in the right cerebellum and right superior parietal gyrus. Significantly increased lrFCD were found in the left posterior temporal lobe and right middle and inferior temporal gyrus, and significantly increased gFCD and lFCD were located in the left superior parietal gyrus. In addition, the significant differences in ALFF and FCDs between the GTCS and HCs groups (TFCE, *p* < 0.05) are shown in Figure 1C and Table 3. Compared with the HCs group, the GTCS group had a significantly increased ALFF in the left postcentral gyrus and left lateral orbital gyrus and significantly decreased ALFF in the right cerebellum and superior parietal gyrus. The significantly increased lrFCD was located in the left superior parietal gyrus and significantly decreased lrFCD was located in the right inferolateral remainder of parietal lobe. The significantly increased gFCD was located in the left superior parietal gyrus and significantly decreased gFCD was located in the left inferolateral remainder of parietal lobe. The significantly increased lFCD was located in the right superior parietal gyrus.

**Table 3:** Significant differences of ALFF and FCDs between patients with JME and HCs, patients with GTCS and HC.

**Parameter**	**Brain regions (Harmmers atlas)**	**MNI Coordinates**			**Cluster size**	**Peak *t*-value**
		**X**	**Y**	**Z**		
**JME>HC**						
ALFF	Right precentral gyrus	27	−15	63	1,556	7.6361
	Left precentral gyrus	−24	−15	63	296	5.6764
	Right superior parietal gyrus	12	−66	36	2,089	−7.7847
	Right cerebellum	6	−57	−54	1,459	−6.3976
lrFCD	Left posterior temporal lobe	−33	−33	−3	965	7.4777
	Right middle and inferior temporal gyrus	42	−24	−21	519	6.7156
	Left superior parietal gyrus	−9	−54	21	208	−4.3469
gFCD	Left superior parietal gyrus	−15	−42	51	60	5.6151
	Right inferolateral remainder of parietal lobe	51	−63	24	59	−4.0931
lFCD	Left superior parietal gyrus	−15	−42	51	366	6.0595
**GTCS>HC**						
ALFF	Left postcentral gyrus	−45	−21	54	651	6.6488
	Left lateral orbital gyrus	−33	36	−9	1186	6.2284
	Right superior parietal gyrus	9	−66	36	2,659	−7.5734
	Right cerebellum	54	−63	−33	460	−5.4859
lrFCD	Left superior parietal gyrus	−18	−42	57	409	4.673
	Right inferolateral remainder of parietal lobe	54	−66	24	76	−4.5046
gFCD	Left superior parietal gyrus	−3	−57	63	375	5.1948
	Left inferolateral remainder of parietal lobe	−42	−63	30	98	−3.9728
lFCD	Right superior parietal gyrus	6	−51	57	251	4.5439

*MNI, Montreal Neurological Institute*.

**Figure 2:**
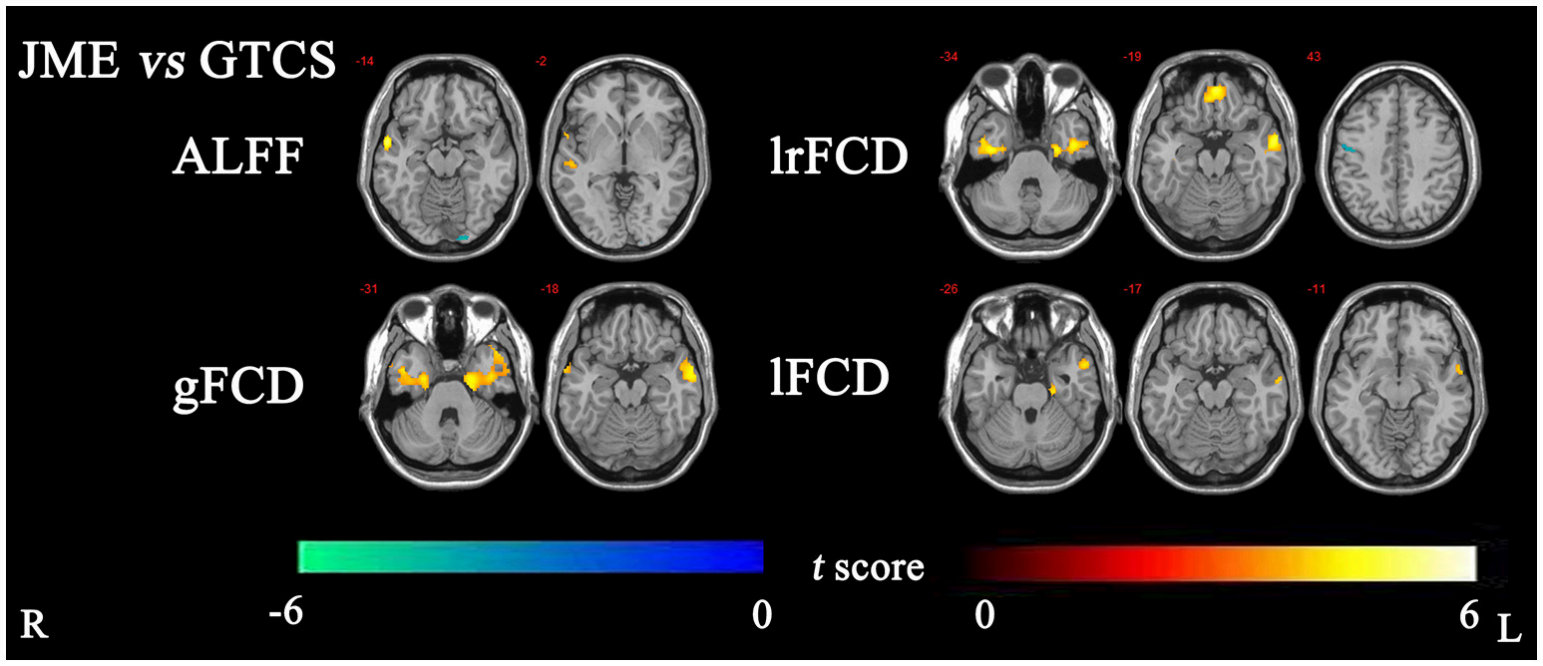
The significant differences of ALFF and FCDs between JME and GTCS (Threshold-Free Cluster Enhancement, *p* < 0.05). The significant increased regions were showed with positive *t*-value and significant decreased regions with negative *t*-value. R, Right; L, Left.

The significant differences in ALFF and FCDs between the JME and GTCS groups (TFCE, *p* < 0.05) are shown in Figure 2 and Table 4. Compared with the GTCS group, the JME group had significantly increased ALFF in left superior temporal gyrus of posterior part. The significantly increased lrFCD were located in the left medial orbital gyrus, left middle and inferior temporal gyrus and right fusiform gyrus and significantly decreased lrFCD was located in the right precentral gyrus. The significantly increased gFCD were located in the left parahippocampal and ambient gyri and right middle and inferior temporal gyrus. The significantly increased lFCD was located in the left anterior temporal lobe of lateral part.

**Table 4:** Significant differences of ALFF and FCDs between patients with JME and GTCS.

**Parameter**	**Brain regions (Harmmers atlas)**	**MNI coordinates**			**Cluster size**	**Peak *t*-value**
		**X**	**Y**	**Z**		
**JME>GTCS**						
ALFF	Left superior temporal gyrus, posterior part	−57	0	−15	83	5.5433
lrFCD	Left medial orbital gyrus	−3	48	−15	295	5.8404
	Left middle and inferior temporal gyrus	−57	−3	−24	469	5.1236
	Right fusiform gyrus	39	−12	−36	195	4.7339
	Right precentral gyrus	42	−12	42	63	−4.3247
gFCD	Left parahippocampal and ambient gyri	−21	−21	−30	701	5.328
	Right middle and inferior temporal gyrus	63	3	−27	243	4.8044
lFCD	Left anterior temporal lobe, lateral part	−54	6	−27	152	4.4284

*MNI, Montreal Neurological Institute*.

### Altered coupling between ALFF and FCDs

The subject-wise correlations are shown in Table 5. Compared with the HCs group, significant differences in coupling of ALFF and lrFCD were found in IGE (*p* = 9.65 × 10^−5^), JME (*p* = 3.30 × 10^−4^), and GTCS (*p* = 0.0087) patients. Meanwhile, no significant differences in the coupling of ALFF and gFCD or ALFF and lFCD were found in patients with IGE, JME, and GTCS.

**Table 5:** The correlation of ALFF and FCDs (lrFCD, gFCD, lFCD) in subject-wise.

**Correlation**	**IGE** **(*n* = 60)** **Mean ± SD**	**JME** **(n = 32)** **Mean±SD**	**GTCS** **(*n* = 28)** **Mean±SD**	**HC** **(*n* = 60)** **Mean±SD**	**P1**	**P2**	**P3**	**P4**
ALFF-lrFCD	0.39 ± 0.11	0.37 ± 0.11	0.40 ± 0.10	0.47 ± 0.13	9.65 × 10^−5^*^^	3.30 × 10^−4^*^^	0.0087^*^	0.3267
ALFF-gFCD	0.53 ± 0.10	0.53 ± 0.11	0.52 ± 0.12	0.56 ± 0.10	0.1127	0.1690	0.1268	0.7954
ALFF-lFCD	0.48 ± 0.11	0.48 ± 0.12	0.47 ± 0.11	0.48 ± 0.10	0.7224	0.8182	0.9643	0.8231

P1 represent the test results of IGE and HC.

P2 represent the test results of JME and HC.

P3 represent the test results of GTCS and HC.

P4 represent the test results of GTCS and JME.

* *There was significant difference of coupling between ALFF and FCDs between patients and HCs (p-value obtained by two-sample t-test of correlation coefficient which Fisher's r-to-z transformed)*.

**Figure 3:**
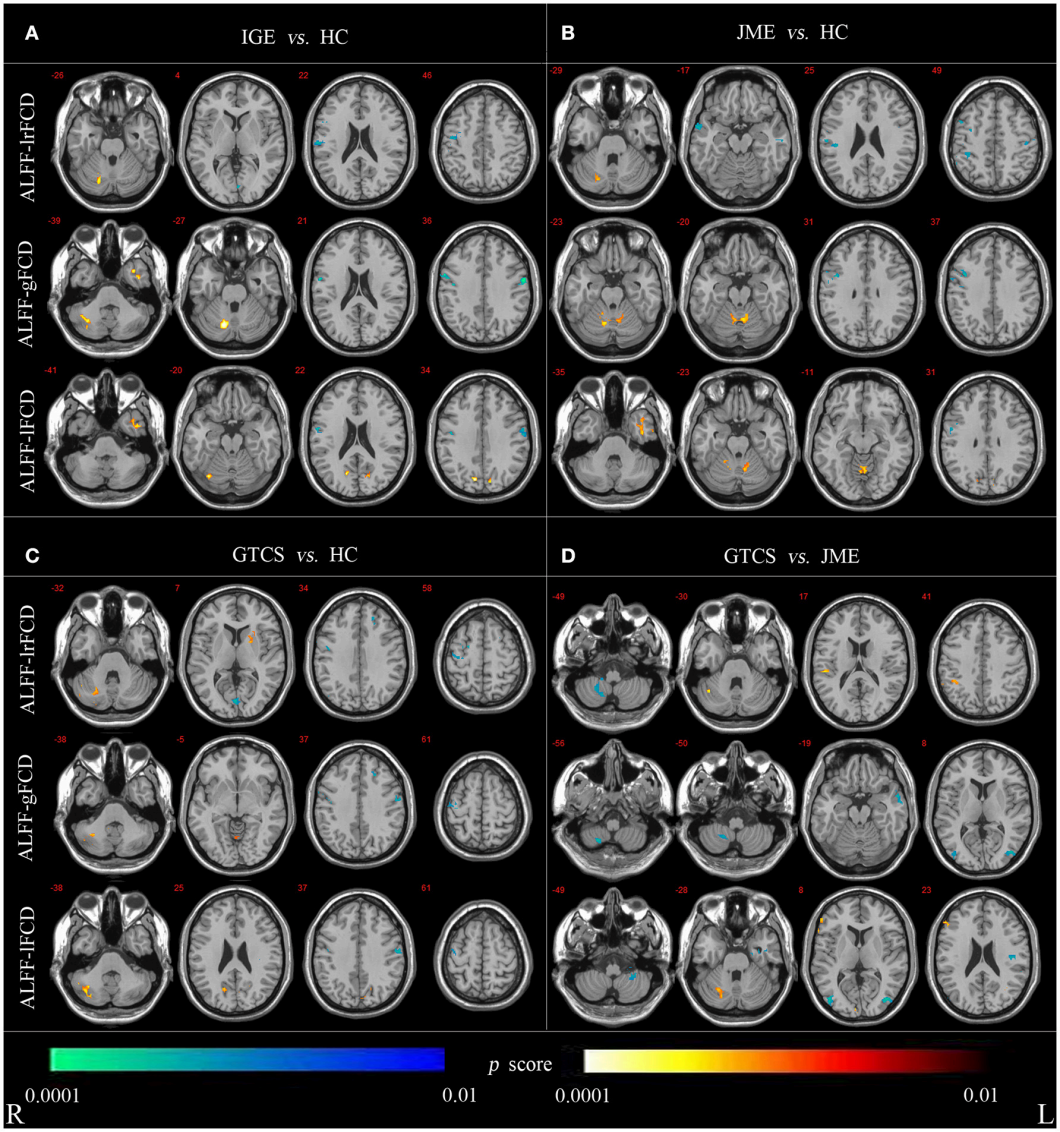
**(A)** Statistic p-map demonstrating the significant difference between the IGE and HC**. (B)** Statistic p-map demonstrating the significant difference between the JME and HC. **(C)** Statistic *p*-map demonstrating the significant difference between the GTCS and HC**. (D)** Statistic *p*-map demonstrating the significant difference between the GTCS and JME (non-parametric permutation test, *p* < 0.01). The hot color reflects increased value and cool color represents decreased value in contrasts IGE vs. HC, GTCS vs. JME, JME vs. HC, GTCS vs. JME. R, Right; L, Left.

For the voxel-wise correlations, all groups showed positive correlations in the cerebellum and cortical structures as shown in Supplementary Figure 2. The significant differences in coupling of ALFF and FCDs between patients (in the IGE, JME, and GTCS groups) and HCs and between patients in the GTCS and JME groups are shown in Figure 3 and Supplementary Table 3.

The non-parametric permutation test with 10,000 repetitions, with voxel-wise *p* < 0.01, to assess coupling of ALFF and FCDs. For coupling of ALFF and lrFCD, patients with IGE (JME and GTCS) showed significantly increased coupling in the cerebellum and significantly decreased coupling in the right precentral and postcentral gyrus compared with the HCs. Significantly decreased coupling in the right lateral remainder of occipital lobe was revealed in patients with IGE, significantly decreased coupling in the right anterior temporal lobe of lateral part was revealed in patients with JME and a significantly increased correlation in the right putamen was obtained in patients with GTCS. Compared with patients in the JME group, patients in the GTCS group showed significantly increased coupling in the right cerebellum, right inferolateral remainder of parietal lobe and significantly decreased coupling in the right cerebellum. For coupling of ALFF and gFCD, the IGE group had significantly increased coupling in the right cerebellum and left anterior temporal lobe of lateral part and significantly decreased coupling in the right precentral gyrus and postcentral gyrus compared with the coupling in the HCs group. The JME group showed significantly increased coupling in the right cerebellum and significantly decreased coupling in the right precentral gyrus. In addition, significantly increased coupling was revealed in the cerebellum and left lingual gyrus, and significantly decreased coupling was revealed in the left middle frontal lobe and bilateral precentral gyrus in the GTCS group. Compared with the JME group, the GTCS group had significantly decreased coupling in the right cerebellum, left middle and inferior temporal gyrus and bilateral lateral remainder of occipital lobe. For coupling of ALFF and lFCD, the IGE group exhibited significantly increased coupling in the right cerebellum, left anterior temporal lobe of medial part and right lateral remainder of occipital lobe and significantly decreased coupling in the right postcentral gyrus. The JME group showed significantly increased coupling in the left cerebellum and right anterior temporal lobe of lateral part and significantly decreased coupling in the right precentral gyrus. In addition, the GTCS group showed significantly increased coupling in the cerebellum and right cuneus and significantly decreased coupling in the right precentral and left postcentral gyrus. Compared with the JME group, the GTCS group showed significantly increased coupling in the right cerebellum and significantly decreased coupling in the left postcentral gyrus, left cerebellum and right lateral remainder of occipital lobe.

### Correlation analysis

**Figure 4:**
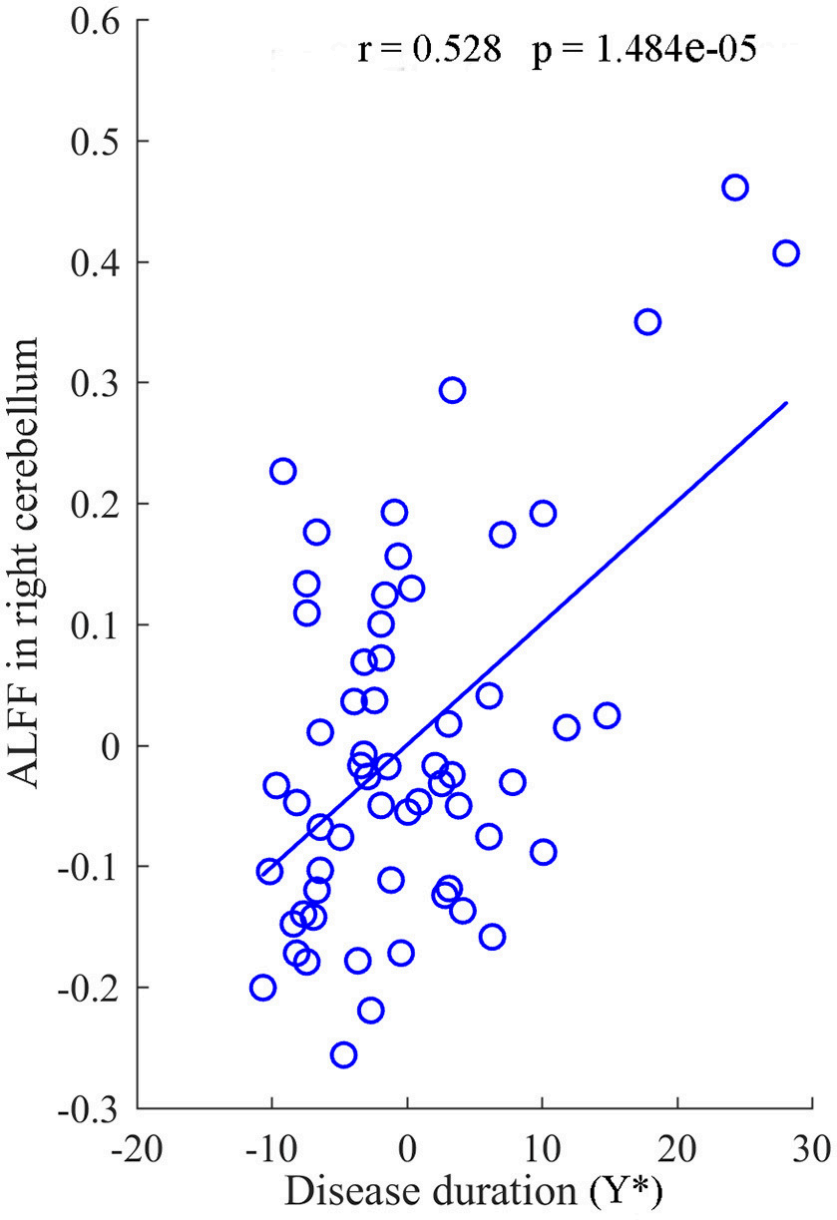
The correlation between the value of ALFF in right cerebellum and the duration of disease for IGE. Y* represent the disease duration years after regressed out the age of onset.

After regressed out the age of onset, a significant positive correlation between the mean value of ALFF in the right cerebellum and the duration of disease was revealed in the IGE group (*R* = 0.528, *P* = 1.484 × 10^−5^) (Figure 4). In addition, there was no significant correlations between other clinical data and the value of ALFF and FCDs in neither the JME nor the GTCS groups.

## Discussion

In the current study, we combined analyses of FCDs and ALFF to comprehensively evaluate the alteration of resting-state spontaneous brain activity in patients with IGE and its subgroups. In our study, patients with IGE, including GTCS and JME, showed reduced ALFF in cerebellum and regions in the DMN (primarily in the posterior part) and increased ALFF in the frontal regions related to motor function. Interestingly, decreased gFCD and lrFCD were also observed in regions of the DMN in patients with IGE, suggesting disruption in the DMN with multifactorial abnormality in IGE. Moreover, increased coupling between FCDs and ALFF was found in the cerebellum, while decreased coupling was observed in the bilateral pre- and postcentral gyrus in IGE compared with coupling in the HCs group. Similar findings were also found in the two subgroups of patients compared with HCs. Importantly, when the comparison was performed between the two subgroups of patients, the decreased coupling was found in the cerebellum and the bilateral middle occipital gyrus in the GTCS group compared with coupling in the JME group. It could be emphasized that the abnormality in the cerebellum not only was associated with dysfunctional motor symptoms during seizures but also might participate in the regulation of GSWDs in IGE. However, no significant difference was observed between the two subgroups of IGE. These results may provide new insights into understanding the pathophysiological mechanisms in patients with IGE.

Consistent with previous studies, an abnormality of the DMN was observed in patients with IGE in this study. In epilepsy, previous studies showed deactivation in the DMN during GSWDs, suggesting that GSWDs may suspend normal function of the brain DMN (6, 33, 34). In addition, the functional connectivity in the DMN was abnormal in IGE even when GSWDs are not present. In this study, we provided more information using BOLD signals and functional connectivity to support an abnormality of the DMN in IGE. Moreover, the alteration of the DMN was similar across variable seizure phenotypes (35). Combined with co-alterations of ALFF and FCDs within the DMN regions in patients, we speculated that the abnormality in the DMN might suggest disturbances of fundamental brain function in patients with IGE and its subgroup. This view was also verified by the similar DMN alternations with brain disorders such as schizophrenia and Parkinson's disease (36–38).

Motor manifestations are observed in some types of generalized seizures, such as tonic-clonic and myoclonic seizures. Consistent with this view, we found increased ALFF and FCDs in the pre- and postcentral gyrus and paracentral lobule in IGE patients, both JME and GTCS patients, who have serious motor symptoms during seizures. Moreover, this increase was shown in the two subgroups of patients when compared with the HCs group separately. The abnormal enhancement of the BOLD signal and functional connectivity in primary sensorimotor cortex is correlated with the motor manifestations in patients with JME and GTCS. Consistent with this finding, the structural and functional abnormalities were observed in previous studies (39–41). For example, the increased local spontaneous brain activation in the precentral gyrus has been found in epilepsy (42). Similarly, using EEG-fMRI, GSWD-related activations in motor-related regions were observed in IGE (43). In addition, the previous study also showed that tonic-clonic and myoclonic seizures were most markedly reduced by biofeedback training of the sensorimotor electroencephalogram rhythm in man (44). More interestingly, it was also suggested that the somatosensory cortex might take part in the generation of GSWDs in IGE (45, 46).

Interestingly, the decreased coupling of ALFF and FCDs in primary sensorimotor cortex was also illustrated in patients, and this decreased coupling may be responsible for the dysfunction of executing voluntary movement in patients with IGE (47). Apart from the primary cortex in the motor system, the decreased ALFF and abnormal coupling of ALFF and FCDs in the cerebellum were observed in patients with IGE in this study. In general, the cerebellum has been considered to play an important role in motor control, such as coordination, precision and accurate timing (48). A clear higher activation in the cerebellum in patients with IGE was found in a previous study using EEG-fMRI (34). Thus, the altered coupling in motor-related intrinsic networks might imply multi-levels abnormality related to motor function in IGE. In addition, the cerebellum may be associated with the regulation of epileptic discharges in IGE (8). In previous EEG-fMRI studies, patients with IGE showed significant activation related to GSWDs in the cerebellum (6, 34). Moreover, the output of cerebellar neurons might regulate spike-and-wave discharges, opening a window for the development of potential treatments for epilepsy, such as deep brain stimulation (49, 50). Thus, our findings of a significantly decreased ALFF in patients with IGE and its subgroups may reflect motor dysfunction, especially myoclonic seizures. Recent studies have suggested that rhythmic output from cerebellum may contribute to the maintenance of generalized petit mal seizures and combination of EEG and fMRI have found activation of cerebellum related to GSWD (51–53). Therefore, to some extent, we presume that the functional alteration in the cerebellum could reflect the contribution effects of cerebellar to the GSWDs in IGE.

Importantly, the coupling of ALFF and FCDs in patients was further analyzed at a subject-wise level. Compared with people in the HCs group, patients with IGE, JME and GTCS had significant decreases in coupling of ALFF and lrFCD. The tight coupling between ALFF and FCDs had been demonstrated in the healthy physiological state (54). Therefore, the decreased coupling of ALFF and lrFCD may reflect pathological uncoupling in patients with IGE and its subgroups.

In summary, we assessed the resting-state fMRI alterations in ALFF and FCDs in patients with IGE. Furthermore, ALFF and FCDs were combined to comprehensively evaluate the altered features of resting-state fMRI in IGE. The abnormality of the DMN obtained using features of BOLD signals and functional connectivity was found in both subgroups of IGE. In addition, the changes of coupling between ALFF and FCDs in the cerebellum and motor-related cortex might be important in IGE, and these findings contribute to a deeper understanding of the motor symptoms and GTCS regulation in IGE.

### Limitations and suggestions for future

Our study still had several limitations. First, relatively small samples of patients were enrolled in our study, which might reduce the reliability of the findings. Finally, it has been suggested that the global signal could obscure underlying neuroanatomical relationships and should be removed (55). Because the global signal might reflect the state of patients with neuro-psychiatric disorders such as schizophrenia (56) and epilepsy (57, 58), the effect of removing the global signal on FC remains unknown. In this study, inclusion of global signal might have affected the findings. In future, the abnormal functional and causal connectivity of IGE at the network-level, such as large-scale network should be considered. In particularly, default mode network and cerebellum should be analyzed according to present results.

## Author contributions

XJ, CL, and SJ generated the idea of this study. SM, LY, and QZ diagnosed patients as IGE and its subgroups (JME and GTCS), interpreted pathology data. XJ, SJ, HS, and XC designed this study, finished the calculation, and interpreted imaging data. XJ and CL drafted the manuscript. XJ, CL, SJ, DY, and DD revised the manuscript.

### Conflict of interest statement

The authors declare that the research was conducted in the absence of any commercial or financial relationships that could be construed as a potential conflict of interest.

## Supplementary material

Supplementary Figure 1 The ALFF and FCDs results for patients with IGE and its subtypes(JME and GTCS) and healthy control. The *T*-values of one-sample *t*-test were showed in each group, respectively. R, Right; L, Left.

Supplementary Figure 2 The results of coupling between ALFF and FCDs on voxel-wise for patients with IGE and its subtypes (JME and GTCS) and healthy control. R, Right; L, Left.

Supplementary Table 1 Clinical information about antiepileptic drugs of GTCS and JME.

Supplementary Table 2 Clinical information about seizure frequency of GTCS and JME.

Supplementary Table 3 The significant differences of coupling between IGE and HC, GTCS and JME, JME and HC, GTCS and HC.
