# Supplementary figures and images for: Disrupted Coupling Between the Spontaneous Fluctuation and Functional Connectivity in Idiopathic Generalized Epilepsy
Source: Front Neurol. 2018 Oct 5;9:838. doi: 10.3389/fneur.2018.00838 (PMC6182059; doi:10.3389/fneur.2018.00838)

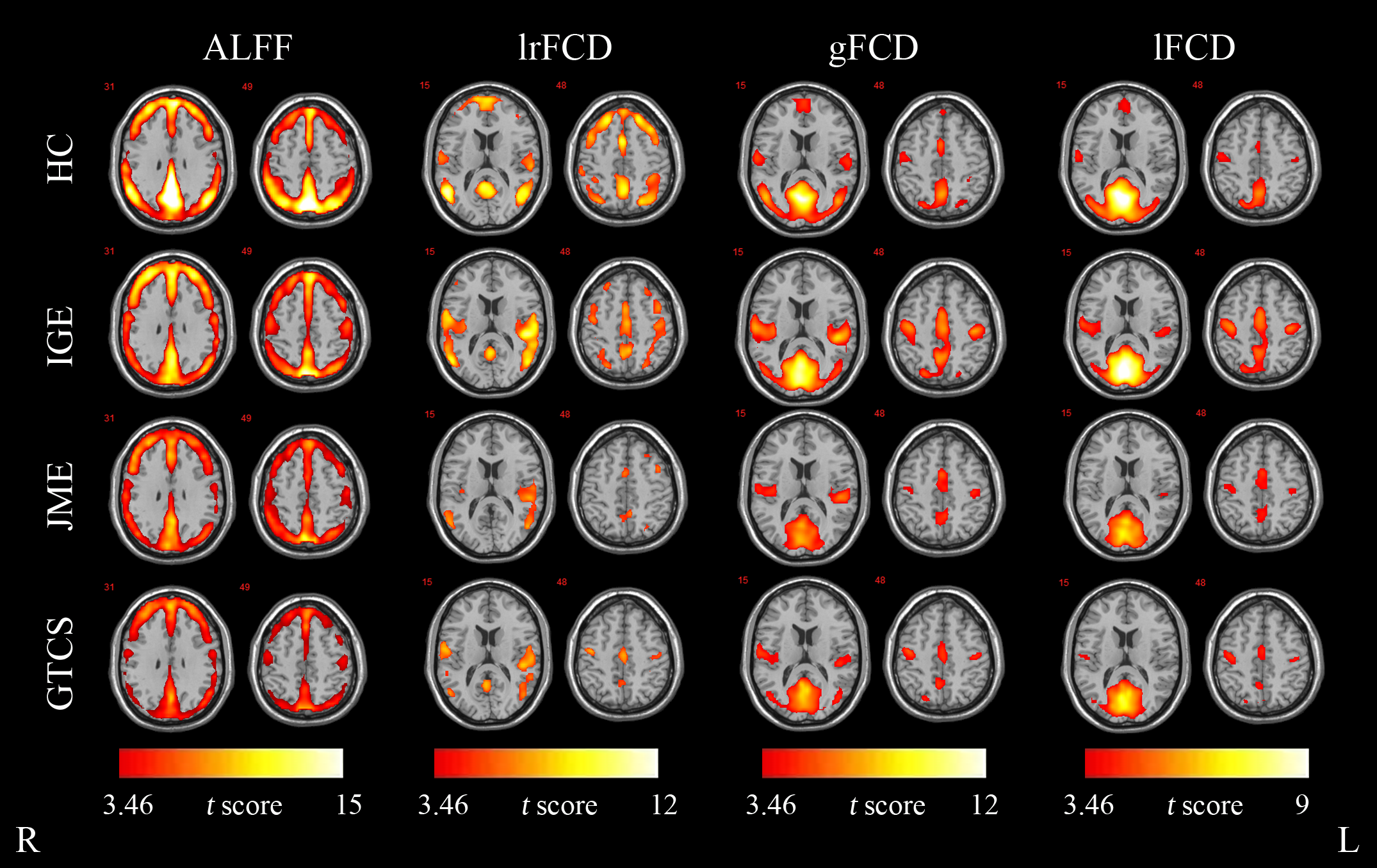

Supplement: Supplementary Figure 1 — The ALFF and FCDs results for patients with IGE and its subtypes(JME and GTCS) and healthy control. The T-values of one-sample t-test were showed in each group, respectively. R, Right; L, Left. [file Image_1.TIF]

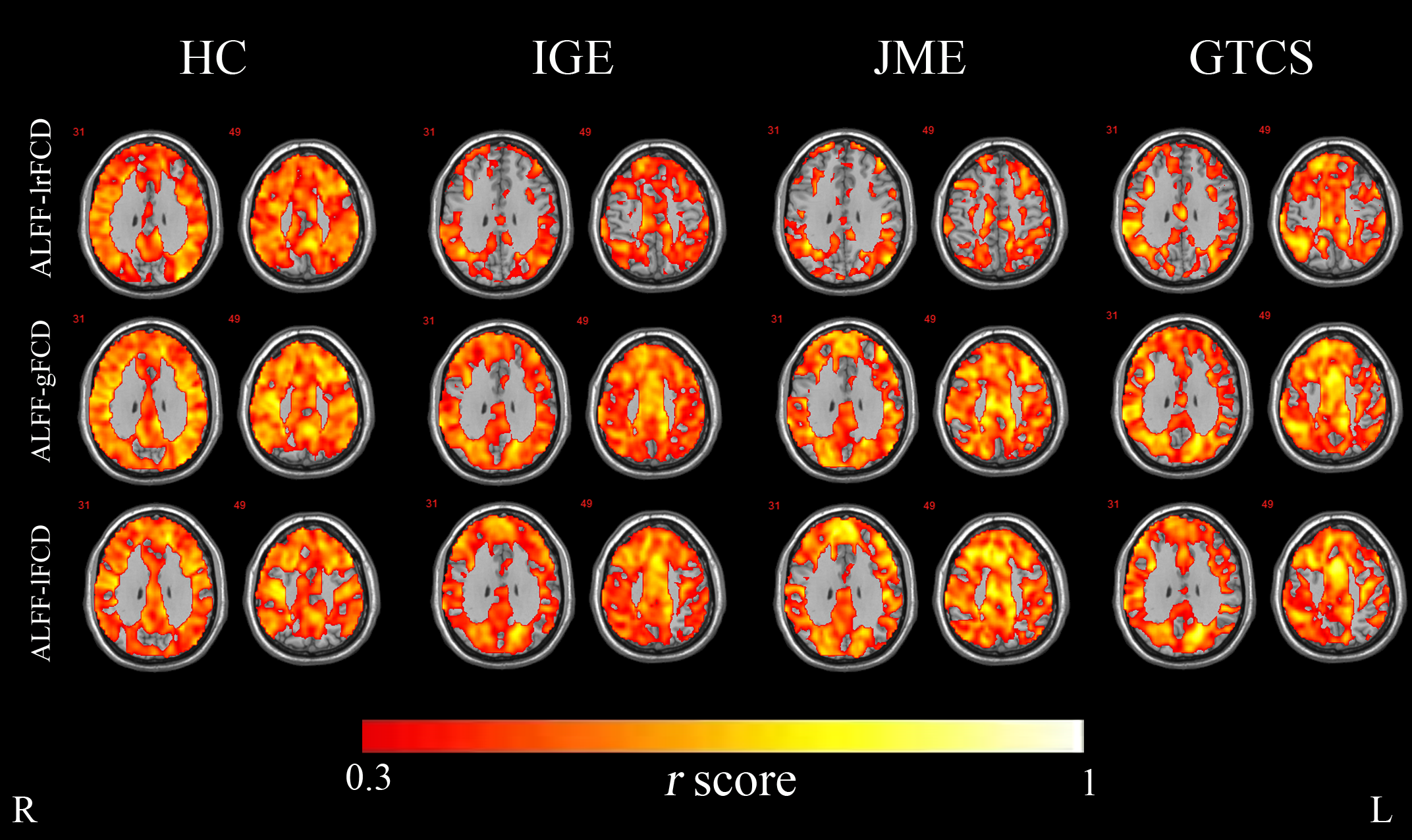

Supplement: Supplementary Figure 2 — The results of coupling between ALFF and FCDs on voxel-wise for patients with IGE and its subtypes (JME and GTCS) and healthy control. R, Right; L, Left. [file Image_2.TIF]
