# Supplementary material for: Disrupted Coupling Between the Spontaneous Fluctuation and Functional Connectivity in Idiopathic Generalized Epilepsy
Source: Front Neurol. 2018 Oct 5;9:838. doi: 10.3389/fneur.2018.00838 (PMC6182059; doi:10.3389/fneur.2018.00838)
Supplement: Supplementary Table 1 — Clinical information about antiepileptic drugs of GTCS and JME. [file Table_1.DOCX]

Supplementary Table 1

: Clinical information about antiepileptic drugs of GTCS and JME.

| Antiepileptic drugs | GTCS (n=28) | JME (n=32) |
| --- | --- | --- |
| LEV | 14 | 1 |
| VAL | 0 | 7 |
| OXC | 1 | 0 |
| VPA | 3 | 14 |
| LTG | 0 | 2 |
| MAG | 0 | 2 |
| CAR | 1 | 0 |
| LEV & TPM | 1 | 0 |
| LEV & LTG | 3 | 0 |
| LEV & VPA | 1 | 1 |
| LTG & VAL | 0 | 2 |
| LEV & CAR | 0 | 1 |
| VPA & CAR | 0 | 1 |
| OXC & VPA | 1 | 0 |
| OXC & TPM | 0 | 1 |
| LEV & OXC & LTG | 1 | 0 |
| LEV & OXC & VPA | 1 | 0 |
| LEV & VPA & LTG | 1 | 0 |

LEV : levetiracetam table; OXC : oxcarbazepine; VPA : valproic acid; LTG : lamotrigine; TPM :Topamax; CAR : carbamazepine; VAL : Valpromide; MAG : Magnesium Valproate;
