# Supplementary material for: Disrupted Coupling Between the Spontaneous Fluctuation and Functional Connectivity in Idiopathic Generalized Epilepsy
Source: Front Neurol. 2018 Oct 5;9:838. doi: 10.3389/fneur.2018.00838 (PMC6182059; doi:10.3389/fneur.2018.00838)
Supplement: Supplementary Table 2 — Clinical information about seizure frequency of GTCS and JME. [file Table_2.DOCX]

Supplementary Table 2

: Clinical information about seizure frequency of GTCS and JME.

| Seizure frequency | GTCS (n=28) | JME (n=32) |
| --- | --- | --- |
| <1 times / year | 9 | 0 |
| 1 times / year | 7 | 2 |
| 2 times / year | 6 | 0 |
| 2~3 times / year | 4 | 0 |
| 4~6 times / year | 2 | 8 |
| 1 times / month | 0 | 11 |
| 2~3 times / month | 0 | 6 |
| 4~5 times / month | 0 | 3 |
| >5 times / month | 0 | 2 |
