# Supplementary material for: Disrupted Coupling Between the Spontaneous Fluctuation and Functional Connectivity in Idiopathic Generalized Epilepsy
Source: Front Neurol. 2018 Oct 5;9:838. doi: 10.3389/fneur.2018.00838 (PMC6182059; doi:10.3389/fneur.2018.00838)
Supplement: Supplementary Table 3 — The significant differences of coupling between IGE and HC, GTCS and JME, JME and HC, GTCS and HC. [file Table_3.DOCX]

Supplementary Table 3: The significant differences of coupling between IGE and HC, GTCS and JME, JME and HC, GTCS and HC.

| Parameter | Brain regions | MNI coordinates | Cluster size | Peak *p* value |
| --- | --- | --- | --- | --- |
|  |  | X Y Z |  |  |
| IGE$\mathbf{>}$HC |  |  |  |  |
| ALFF-lrFCD | Right cerebellum | 17 -70 -26 | 70 | 0.0009 |
|  | Right postcentral gyrus | 60 -16 23 | 77 | -0.0009 |
|  | Right precentral gyrus | 48 2 32 | 120 | -0.001 |
|  | Right lateral remainder of occipital lobe | 21 -75 11 | 34 | -0.0045 |
| ALFF-gFCD | Right cerebellum | 13 -70 -28 | 155 | 0.0001 |
|  | Left anterior temporal lobe, lateral part | -44 1 -40 | 31 | 0.0002 |
|  | Right postcentral gyrus | 60 -5 35 | 40 | -0.0001 |
|  | Right precentral gyrus | 50 -5 23 | 100 | -0.0004 |
| ALFF-lFCD | Right superior parietal gyrus | 19 -64 29 | 81 | 0.0001 |
|  | Right lateral remainder of occipital lobe | 33 -75 -18 | 45 | 0.0001 |
|  | Left anterior temporal lobe, medial part | -38 9 -40 | 55 | 0.0009 |
|  | Right precentral gyrus | 56 -3 20 | 65 | -0.0009 |
| JME$\mathbf{>}$HC |  |  |  |  |
| ALFF-lrFCD | Right cerebellum | 13 -70 -34 | 160 | 0.0019 |
|  | Left postcentral gyrus | -48 -20 50 | 83 | -0.001 |
|  | Right anterior temporal lobe, lateral part | 57 4 -18 | 65 | -0.0027 |
|  | Right Middle frontal gyrus | 43 14 32 | 658 | - 0.0029 |
| ALFF-gFCD | Right cerebellum | 10 -63 -24 | 390 | 0.001 |
|  | Right precentral gyrus | 54 -3 26 | 230 | -0.0042 |
| ALFF-lFCD | Left cerebellum | -8 -60 -13 | 92 | 0.001 |
|  | Left cerebellum | -11 -56 -21 | 103 | 0.0015 |
|  | Right anterior temporal lobe, lateral part | 42 14 -37 | 142 | 0.0017 |
|  | Right precentral gyrus | 48 -1 30 | 65 | -0.0018 |
| GTCS$\mathbf{>}$HC |  |  |  |  |
| ALFF-lrFCD | Right cerebellum | 22 -64 -31 | 268 | 0.0019 |
|  | Left putamen | -24 5 8 | 263 | 0.0024 |
|  | Right cuneus | 3 -79 8 | 112 | -0.001 |
|  | Right precentral gyrus | 34 -15 56 | 112 | -0.0015 |
|  | Left middle frontal gyrus | -26 30 35 | 70 | -0.0045 |
| ALFF-gFCD | Right cerebellum | 12 -66 -37 | 761 | 0.001 |
|  | Left lingual gyrus | -4 -61 -4 | 226 | 0.001 |
|  | Left precentral gyrus | -59 -3 35 | 71 | -0.001 |
|  | Right precentral gyrus | 42 -12 59 | 90 | -0.0032 |
|  | Left middle frontal gyrus | -24 31 35 | 71 | -0.0042 |
| ALFF-lFCD | Right cerebellum | 36 -68 -39 | 726 | 0.0015 |
|  | Right cuneus | 10 -67 23 | 65 | 0.0019 |
|  | Right precentral gyrus | 39 -14 62 | 136 | -0.0013 |
|  | Left postcentral gyrus | -62 -11 36 | 149 | -0.0021 |
| GTCS$\mathbf{>}$JME |  |  |  |  |
| ALFF-lrFCD | Right cerebellum | 37 -56 -30 | 62 | 0.0009 |
|  | Right inferolateral remainder of parietal lobe | 46 -28 17 | 52 | 0.002 |
|  | Right inferolateral remainder of parietal lobe | 38 -43 41 | 43 | 0.0036 |
|  | Right cerebellum | 21 -52 -49 | 111 | -0.0034 |
| ALFF-gFCD | Right cerebellum | 20 -60 -55 | 55 | -0.001 |
|  | Left middle and inferior temporal gyrus | -63 -8 -22 | 64 | -0.0011 |
|  | Left lateral remainder of occipital lobe | -41 -79 8 | 45 | - 0.002 |
|  | Right lateral remainder of occipital lobe | 41 -83 11 | 50 | -0.0039 |
| ALFF-lFCD | Right cerebellum | 25 -65 -25 | 131 | 0.0025 |
|  | Left postcentral gyrus | -44 -16 23 | 51 | - 0.0018 |
|  | Right lateral remainder of occipital lobe | 40 -80 8 | 109 | - 0.0021 |
|  | Right cerebellum | 30 -45 -49 | 104 | - 0.0036 |

MNI: Montreal Neurological Institute;
